# Hypothyroidism Affects Vascularization and Promotes Immune Cells Infiltration into Pancreatic Islets of Female Rabbits

**DOI:** 10.1155/2015/917806

**Published:** 2015-06-14

**Authors:** Julia Rodríguez-Castelán, Margarita Martínez-Gómez, Francisco Castelán, Estela Cuevas

**Affiliations:** ^1^Doctorado en Ciencias Biológicas, Universidad Autónoma de Tlaxcala, 90070 Tlaxcala, TLAX, Mexico; ^2^Centro Tlaxcala de Biología de la Conducta, Universidad Autónoma de Tlaxcala, 90070 Tlaxcala, TLAX, Mexico; ^3^Departamento de Biología Celular y Fisiología, Instituto de Investigaciones Biomédicas, Universidad Nacional Autónoma de México, Unidad Periférica, 90070 Tlaxcala, TLAX, Mexico

## Abstract

Thyroidectomy induces pancreatic edema and immune cells infiltration similarly to that observed in pancreatitis. In spite of the controverted effects of hypothyroidism on serum glucose and insulin concentrations, the number and proliferation of Langerhans islet cells as well as the presence of extracellular matrix are affected depending on the islet size. In this study, we evaluated the effect of methimazole-induced hypothyroidism on the vascularization and immune cells infiltration into islets. A general observation of pancreas was also done. Twelve *Chinchilla*-breed female adult rabbits were divided into control (*n* = 6) and hypothyroid groups (*n* = 6, methimazole, 0.02% in drinking water for 30 days). After the treatment, rabbits were sacrificed and their pancreas was excised, histologically processed, and stained with Periodic Acid-Schiff (PAS) or Masson's Trichrome techniques. Islets were arbitrarily classified into large, medium, and small ones. The external and internal portions of each islet were also identified. Student-*t*-test and Mann-Whitney-*U* test or two-way ANOVAs were used to compare variables between groups. In comparison with control rabbits, hypothyroidism induced a strong infiltration of immune cells and a major presence of collagen and proteoglycans in the interlobular septa. Large islets showed a high vascularization and immune cells infiltration. The present results show that hypothyroidism induces pancreatitis and insulitis.

## 1. Introduction

A stretch relationship between the function of the pancreas and the hypothalamic-pituitary-thyroid axis has been established. The pancreas of animal and human subjects contains high thyrotropin release hormone (TRH) levels. The TRH is located in secretory granules of insulin-containing cells [1], regulates the concentration of pancreatic enzymes, and induces pancreatic hyperplasia [2]. The presence of thyrotropin receptor (TSHR) [3] and thyroid hormones receptors (TRs) *α* and *β* [3] supports that acinar and islet cells are sensitive to thyroid hormones and thyrotropin (TSH). Thyroid hormones participate in the development and proliferation of acinar cells, as well as in the secretion of pancreatic enzymes [3–7]. They also regulate proliferation and survival of islet cells, insulin sensitivity and insulin synthesis [3, 8–10], and the reprogramming of pancreatic acinar cells to insulin-producing cells [11, 12].

Hyperglycemia and insulin resistance are highly related to subclinical and clinical hypothyroidism [13, 14]. The exocrine function of the pancreas seems also to be altered in hypothyroid subjects [15]. Even in euthyroid people, the serum concentrations of TSH, thyroxin (T4), and triiodothyronine (T3) have been directly associated with the serum glucose and insulin concentration, as well as with insulin resistance [16, 17]. In adult animals, thyroidectomy induces interstitial edema and degenerative changes in the pancreatic acinar cells decreasing the number and size of the zymogen granules [18]. In spite of the discrepancy regarding the effect of hypothyroidism on the concentration of glucose and insulin [3, 19–21], this hormonal condition affects the number and proliferation of islet cells and the content of extracellular matrix into the islets [3]. This suggests that histological changes in endocrine pancreas induced by hypothyroidism could occur before insulin and glucose alteration. However, there are scarce studies focused on analyzing the impact of hypothyroidism on the morphohistology of pancreatic islets. Therefore, the present study aimed to evaluate the effect of methimazole-induced hypothyroidism on the general characteristics of the pancreas, particularly on the vascularization and immune cells infiltration into islets of female rabbits.

## 2. Materials and Methods

Twelve* Chinchilla*-breed virgin female rabbits (*Oryctolagus cuniculus*), 9-10 months old, were housed with temperature and artificial illumination controlled (20 ± 2°C; light : dark 06:00 to 22:00 h). Most of females are in early proestrus under these conditions [22]. Control and hypothyroid females were daily provided with pellet food and tap water* ad libitum* for one month. Hypothyroidism was induced by adding 0.02% methimazole (MMI; Sigma; approximate diary dosage 10 mg/kg) to the tap water as described elsewhere [3, 23]. The Guidelines of Mexican Law of Production, Care and Use Laboratory Animals (NOM-062-ZOO-1999) were thoroughly followed along the experimental period.

All rabbits used in the following procedures were euthanized with an overdose of sodium pentobarbital (60 mg/kg). Immediately after death, left lobes of pancreas were fixed in Bouin-Duboscq fixative and histologically processed. Pancreas was embedded in paraplast X-tra (Sigma-Aldrich) and longitudinally cut at a thickness of 5 *μ*m using a microtome (Model 325, Thermo Scientific). Tissue sections were mounted on gelatin-coated slides (Sigma-Aldrich). One section from the central portion of the pancreas per female rabbit was stained with Masson's Trichrome to observe the presence of collagen [24, 25]. Another section was stained with Periodic Acid-Schiff (PAS) and counterstained with Mayer's hematoxylin to detect capillary basement membrane [26, 27] and proteoglycans present in the extracellular matrix of the pancreas [28]. Pictures of pancreas were taken with an optical microscope at 4x (Zeiss Axio Imager A1).

### 2.1. Islets Blood Vessels

The analysis of pancreatic vascularization per female rabbit was done on reconstructed images. Using a grid of 24 × 20 cm on the computer screen, some islets were randomly chosen and photographed at 40x. The cross-sectional area of each islet was measured using Axiovision 4.8 (Carl Zeiss MicroImaging, Inc.), and islets were classified into large, medium, and small ones [3]. The number of blood vessels per islet was obtained by counting all those present inside of islets using the ImageJ 1.43 software. Individual vessels were considered as those separated from others by a space. This variable was measured in the full islet, including the external and internal portions of each. For this last, the diameter of each islet was divided into four parts: the two outer portions were considered as the external portion, while the two inner portions were taken as the internal portion of each islet. For those islets with an irregular form, the shorter diameter was considered in this analysis. The area covered by basement membrane of blood vessels per islet was quantified as the blood vessel wall using the Axiovision program.

### 2.2. Number of Immune Cells into Blood Vessel of Islets

The number of immune cells into blood vessels present in overall islets and in islets classified by size was quantified in randomly chosen islet PAS-stained. Cells observed in the capillary membrane considered as endothelial cells and pericytes were omitted from the counting. The presence of immune cells into blood vessels of the internal and external portions of each islet was also analyzed.

### 2.3. Statistical Analyses

Statistical analyses were performed with the GraphPad Prism v5.01 program (GraphPad Software, Inc., CA, USA). Results were expressed as mean ± SEM for each value. Student's *t*-test or Mann-Whitney *U*  test was used to determine significant differences between control and hypothyroid rabbits. Differences between groups considering the size of the islets were compared by two-way ANOVAs and Newman's post hoc test. The values of *P* < 0.05 were considered statistically significant.

## 3. Results

**Figure 1:**
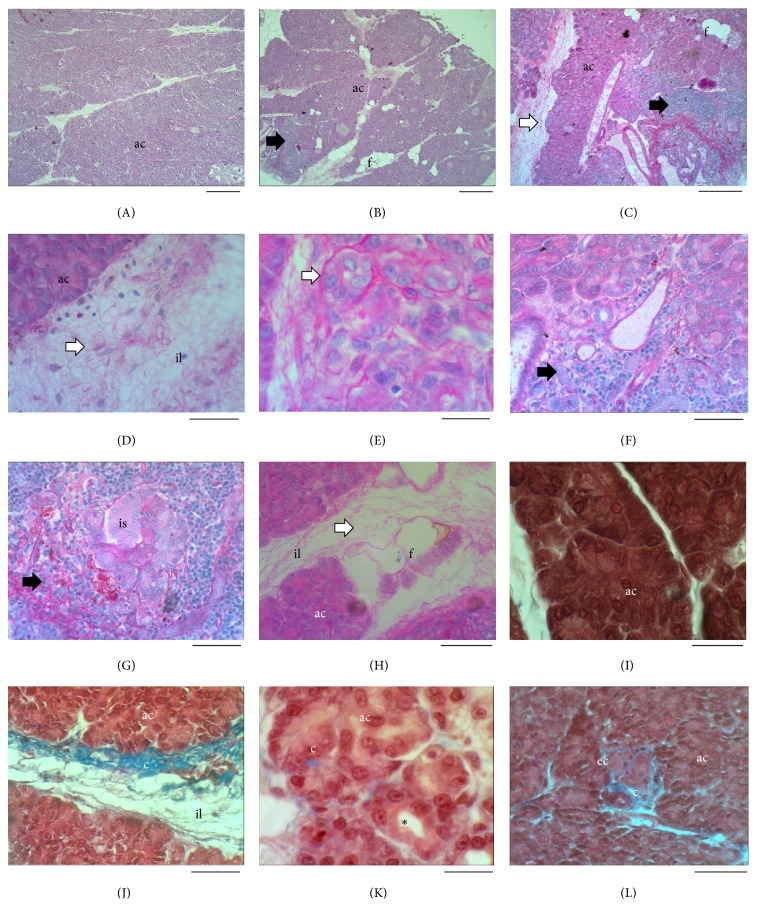
Histological characteristics of the pancreas from control and hypothyroid female rabbits. Pictures of pancreas from control ((A) and (I)) and hypothyroid ((B)–(H) and (J)–(L)) females, showing the presence of proteoglycans ((C)–(H)) as a component of the extracellular matrix, the infiltration of immune cells ((B)–(G)), and the presence of collagen ((I)–(L)). Bar: (A)-(B) = 200 *μ*m; (C) = 100 *μ*m; (D), (F)–(H), (J), and (L) = 50 *μ*m; and (E), (I), and (K) = 20 *μ*m. ac: acinar cells; is: islets; il: interlobular septa; c: collagen; f: fat; ec: stellate cells. Black arrow: immune cells infiltration; white arrow: proteoglycans; asterisk: metaplasia.

In comparison with the control group (Figure 1(A)), pancreas from hypothyroid females showed a high presence of white adipocytes (Figures 1(B) and 1(H)). Fibers of proteoglycans were located among the interlobular septa (Figures 1(C)–1(E) and 1(H)) and surrounding acinar cells (Figure 1(E)). A strong infiltration of immune cells was evident toward acinar tissue and islets (Figures 1(E)–1(G)) in hypothyroid animals. Some groups of cells were observed into the interlobular septa, separated from the acinar cells (Figure 1(H)). The presence of collagen in the interlobular septa and islets was higher in hypothyroid than in control animals (Figures 1(I)–1(K)). Some nodular shapes with cells and collagen were also identified (Figure 1(L)). Independent of the size of islets, the basement membrane of blood vessels was observed in the sections stained with PAS (Figure 1(G)).

**Figure 2:**
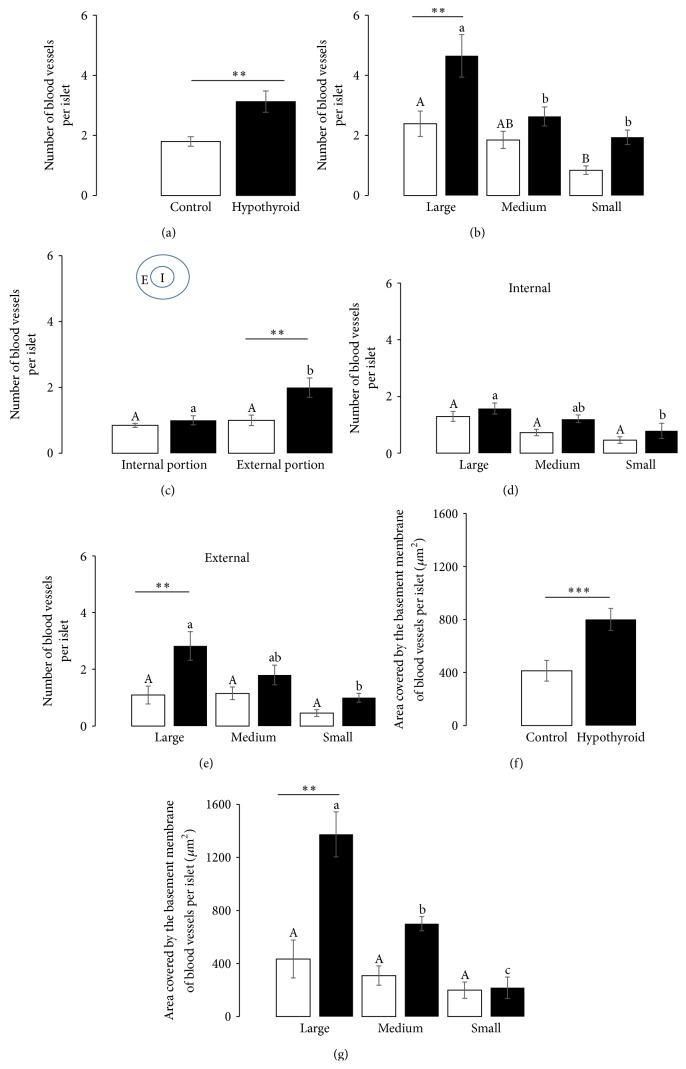
Vascularization of islets from control (*light*) and hypothyroid (*dark*) female rabbits. Number of blood vessels in overall islets (a), in islets categorized by size (b), in portions of the islets (c), in the internal portion of islets categorized by size (d), and in the external portion of islets categorized by size (e). Area covered by the basement membrane of blood vessels for overall islets (f) and categorized by size (g). Different capital letters indicate significant differences between sizes of islets in control females. A similar order is given by lowercase letters for hypothyroid females. Asterisks indicate significant differences between treatments ^*∗∗*^ *P* < 0.01 and ^*∗∗∗*^ *P* < 0.001. E: external portion of the islet; I: internal portion of the islet.

Compared to control rabbits, the number of blood vessels was large in islets from hypothyroid rabbits (Figure 2(a)). The classification of islets by size showed that the number of blood vessels was larger in large islets than in small islets of the control group. Meanwhile, the number of blood vessels was larger in large islets than both medium and small islets of the hypothyroid group (Figure 2(b)). Compared to the control group, the number of blood vessels was only larger in large islets of the hypothyroid group, but not in medium or small ones (Figure 2(b)). No statistical differences were found when blood vessels from internal and external portions of islets of the control group were compared. However, a large number of blood vessels were found at the external portion of islets in the hypothyroid group compared to the control group (Figure 2(c)). To classify islets considering size of islets and the pancreatic portion in which they were located (internal or external), it was noticed that the size of islets did not affect the vascularization of the control group (Figures 2(d) and 2(e)). By contrast, hypothyroidism increased the presence of blood vessels in large islets, only at the external portion (Figure 2(e)). Furthermore, hypothyroidism increased the area covered by the basement membrane of blood vessels per islet as compared to control rabbits (Figure 2(f)). Whereas this variable was not affected by the size of islets in the control group, it was indeed increased in large ones, followed by medium and small islets in hypothyroid females. Noticeably, the area covered by the basement membrane of blood vessels was higher in large islets of hypothyroid rabbits than in those of control rabbits (Figure 2(g)).

**Figure 3:**
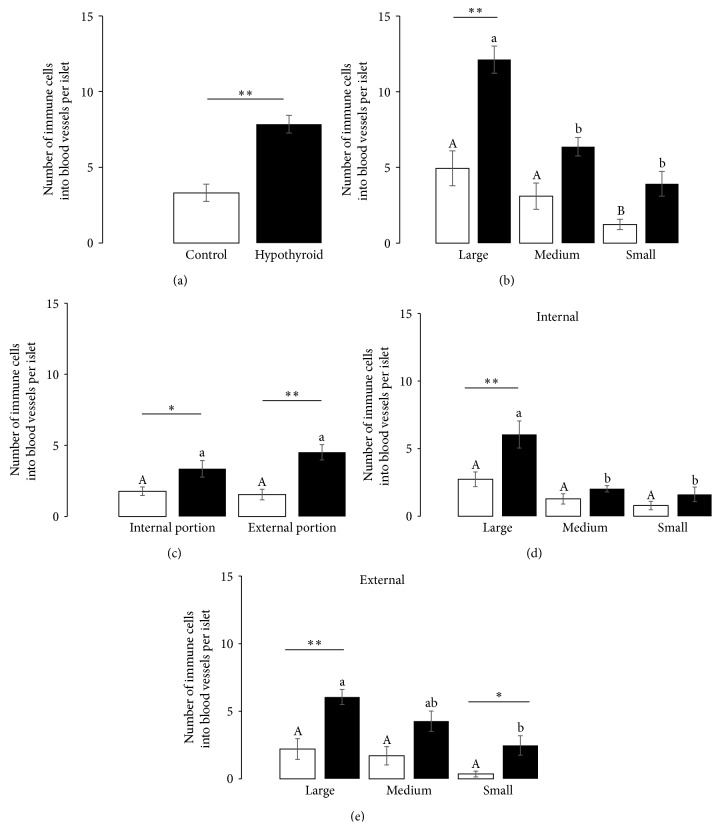
Infiltration of immune cells into islets from control (*light*) and hypothyroid (*dark*) female rabbits. Number of immune cells in overall islets (a), in islets categorized by size (b), in portions of the islets (c), in the internal portion of islets categorized by size (d), and in the external portion of islets categorized by size (e). Different capital letters indicate significant differences between sizes of islets in control females. A similar order is given by lowercase letters for hypothyroid females. Asterisks indicate significant differences between treatments ^*∗*^ *P* < 0.05 and ^*∗∗*^ *P* < 0.01.

Hypothyroidism increased the number of immune cells into blood vessels (Figure 3(a)). In the control group, large and medium size islets had a larger number of immune cells than small ones. In the hypothyroid group, large islets had a larger number of immune cells than medium and small ones. In comparison with the control group, only large islets of hypothyroid females had a large number of immune cells (Figure 3(b)). The number of immune cells into blood vessels was similar at the internal or external portions of islets in the control group. The same was true for the hypothyroid group. However, the number of immune cells at the internal or external portions of islets was larger in the hypothyroid than in the control group (Figure 3(c)). The double classification of the presence of immune cells into islets according to the size and the internal or external portions of islets showed that, independently of the islet size, islets of the control group had a similar number of immune cells at the internal or external portions (Figures 3(d) and 3(e)). In contrast, at both the internal and external portions, large islets had the largest number and the small ones the smaller number of immune cells in the hypothyroid group (Figures 3(d) and 3(e)). At the internal portion, large islets had a larger number of immune cells in the hypothyroid group than control (Figure 3(d)). At the external portion, both large and small islets showed a larger number of immune cells in hypothyroid than in control females (Figure 3(e)).

## 4. Discussion

Our results extend the knowledge about the impact of hypothyroidism on exocrine and endocrine pancreatic tissues. In addition to the presence of adipocytes, an increase of the number of islet cells and the extracellular matrix, and the immunoreactivity antithyroid hormone receptors and TSHR previously described in the female rabbit [3], present findings show that hypothyroidism affects the presence of blood vessels into the islets and support an ongoing inflammatory process in the pancreas.

Methimazole treatment for 1 month increases the amount of collagen and proteoglycans in the interlobular septa and the infiltration of immune cells in the exocrine tissue of the pancreas. Indeed, both the presence of collagen and presence of immune cells in acinar tissue are considered histological indicators of a pancreatitis state [29, 30] and are also presented in diabetic animals [24, 25]. Additionally, proteoglycans present in pancreas could be able to activate immune cells, favoring the production of interleukins [31] and the development of diabetes [32]. Even proteoglycans are associated with the amyloid deposition in cultured human islets [33]. The augmentation of collagen and noncollagenous fibrillary structures in the pancreas has been related to the formation of microcompartments modifying the function of pancreas ducts, blood, and lymphatic vessels [34]. Our findings agree with the effect induced by thyroidectomy in rats, promoting interstitial edema and degenerative changes in the pancreatic acinar cells and decreasing the number and size of zymogen granules [18]. Nevertheless, our present findings are opposed to those obtained in rats with pancreatitis induced by cerulean, where the treatment with methimazole for 10 days has a protective action [35]. In comparison with this last study, the treatment that we used in the present study was long suggesting that chronic hypothyroidism alters the histological characteristics of exocrine pancreas. Although we did not measure the profile of pancreatic enzymes, the pancreatic secretion of both bicarbonate and enzymes is certainly reduced in patients with hypothyroidism compared to healthy subjects [15].

An increase in the blood vessels into large islets was also found. In this regard, the presence of lymphatic vessels has been described in the interlobular septa, but not into islets [36, 37]. In this way, the vessels stained by PAS correspond to blood vessels. Microvasculature plays an important role in the islet function, regulating the islet maturation during the development of the pancreas [38] and affecting the survival and insulin secretion of transplanted islets [39]. However, a great vascularization of islets is associated with macrophage infiltration and proinflammatory cytokine production, resulting in impaired insulin secretion, a decrease in the beta cell mass, and hyperglycemia [39, 40]. The increase of extracellular matrix into islets of hypothyroid animals previously reported [3] could be related to the rise of blood vessels now found, because metalloproteinases have proangiogenic effects participating in the release of vascular endothelial growth factor A (VEGF-A) [41].

We also found that large and small islets contain a large number of immune cells suggesting an inflammatory process. In concordance with our results, insulitis is presented at the beginning of the type 1 diabetes mellitus in children [42], in patients with type 2 diabetes mellitus, and in animals with natural or induced diabetes [24, 43]. Although it has been reported that a great proportion of blood vessels into islets may enhance the accumulation of inflammatory cells [39, 44], having a positive relationship between vascularity and immune cells infiltration, our results suggest that this depends on the size of the islet and the portion of them (internal or external). Large islets might have more space to contain a great amount of vasculature compared to that of small ones and the external portion of islets might contain more blood vessels than the internal one. Thus, mechanisms by which immune cells arrive at the different portions of the islet and size of islets could vary. In large islets, it may be that angiogenesis occurs, while in small ones it might be more reasonable that the capillary dilation occurs [45]. Further studies should be addressed to test this hypothesis. In this regard, other differences between large and small islets have been described; for example, large islets receive blood from one to three afferent arterioles, whereas the capillaries of smaller islets are integrated with the exocrine capillary system [44, 46]. Moreover, small islets secrete more insulin than large islets [47], and small islets are more resistant to the impact of hypothyroidism on their morphometric characteristics because of their high proliferation [3].

In spite of the hypervascularization and immune cells infiltration in pancreas of female rabbits, we previously showed that neither insulin nor glucose levels are affected by hypothyroidism [3]. It is probable that a long time of treatment with methimazole may result in hyperglycemia and hyperinsulinemia because the treatment with thyroid hormones reduces the hyperglycemia of diabetic rats [48]. Thus, our present findings (inflammation, changes in vascularization, and immune cells infiltration into the islets) could appear before consolidating hyperinsulinemia and a hyperglycemic status. Although we did not identify the type of islet cells, it is possible that insulitis could diminish the number of beta cells as what occurs in patients with type 2 diabetes [49]. Further studies are necessary to elucidate the implication of vascular and immunity changes promoted by hypothyroidism on the function of islets.

Our findings allow concluding that hypothyroidism induces pancreatitis and insulitis. It affects the extracellular matrix and infiltration of immune cells into the acinar tissue. Moreover, hypothyroidism affects differentially the vascularization and immune infiltration of islets according to their size.
